# The generation and functional characterization of induced pluripotent stem cells from human intervertebral disc nucleus pulposus cells

**DOI:** 10.18632/oncotarget.17446

**Published:** 2017-04-26

**Authors:** Yanxia Zhu, Yuhong Liang, Hongxia Zhu, Cuihong Lian, Liang Wang, Yiwei Wang, Hongsheng Gu, Guangqian Zhou, Xiaoping Yu

**Affiliations:** ^1^ Shenzhen Key Laboratory for Anti-Aging and Regenerative Medicine, Health Science Center, Shenzhen University, Shenzhen, 518060, China; ^2^ Department of Spinal Surgery, Xiaogan Maternity and Child Healthcare Hospital, Xiaogan, 432100, China; ^3^ Burns Research Group, ANZAC Research Institute, University of Sydney, Concord, NSW, 2139, Australia; ^4^ Department of Spinal Surgery, The First Affiliated Hospital of Shenzhen University, Shenzhen, 518060, China

**Keywords:** disc degenerative disease, nucleus pulposus, induced pluripotent stem cell, reprogram, differentiation

## Abstract

Disc degenerative disease (DDD) is believed to originate in the nucleus pulposus (NP) region therefore, it is important to obtain a greater number of active NP cells for the study and therapy of DDD. Human induced pluripotent stem cells (iPSCs) are a powerful tool for modeling the development of DDD in humans, and have the potential to be applied in regenerative medicine. NP cells were isolated from DDD patients following our improved method, and then the primary NP cells were reprogramed into iPSCs with Sendai virus vectors encoding 4 factors. Successful reprogramming of iPSCs was verified by the expression of surface markers and presence of teratoma. Differentiation of iPSCs into NP-like cells was performed in a culture plate or in hydrogel, whereby skin fibroblast derived-iPSCs were used as a control. Results demonstrated that iPSCs derived from NP cells displayed a normal karyotype, expressed pluripotency markers, and formed teratoma in nude mice. NP induction of iPSCs resulted in the expression of NP cell specific matrix proteins and related genes. Non-induced NP derived-iPSCs also showed some NP-like phenotype. Furthermore, NP-derived iPSCs differentiate much better in hydrogel than that in a culture plate. This is a novel method for the generation of iPSCs from NP cells of DDD patients, and we have successfully differentiated these iPSCs into NP-like cells in hydrogel. This method provides a novel treatment of DDD by using patient-specific NP cells in a relatively simple and straightforward manner.

## INTRODUCTION

The healthy intervertebral disc (IVD) relies upon the well hydrated and proteoglycan-rich nucleus pulposus (NP) tissue which supports spinal mobility and distributes joint loading. IVD disorders may contribute to pain and disability in patients and degeneration of the nucleus pulposus (NP) is thought to be the main cause of disc degenerative disease (DDD) [1, 2]. During DDD, NP cells (NPCs) decrease in number and the remaining cells have an altered phenotype that is associated with increased degradation and decreased synthesis of the extracellular matrix [2].

Cell-based tissue regeneration including autologous chondrocytes, primary NPCs and stem cells has great impact for DDD. [3, 4]. Although autologous cells have the advantage of no immune response, the availability of autologous NPCs is extremely low in the adult. Furthermore, due to their lack of proliferation, primary NPC cultures are therefore not satisfactory for disc repair. Mesenchymal stem cells (MSCs) are an alternative method for cellular therapy, however, the differentiation ability of MSCs into NP cells is poor compared to that of embryonic stem cells (ESCs). Moreover, because of ethical issues with ESCs, cell source is very limited for cell-based IVD regeneration.

Induced pluripotent stem cells (iPSCs) alleviate the ethical concerns of ESCs; As it can be reprogrammed from adult tissue cells, iPSCs are a possible autologous source for individualized treatment. iPSCs have been successfully reprogrammed from several human somatic cell types [5–7], however, the methods and cell sources most suitable for iPSC clinical applications remain undetermined. It is now well recognized that iPSCs derived from a patient's own somatic cells provide an attractive cell source, which have the potential to differentiate into various cell types for disease modeling, drug discovery and cell therapy.

However, reprogramming the accessible patient's tissue is inefficient and is limited by barriers such as the stage of differentiation and the age of cells [8]. Most of patients who need individual treatment are elderly people, and since aged cells limit the efficiency and fidelity of reprogramming, it is important to develop a good method for aged-cell reprogramming, especially in the aged diseased tissue cell.

iPSCs generated with retroviral or lentiviral methods carry viral transgene insertions in the host genome, and their safety is still of concern in clinical applications [9, 10]. There are other vectors for iPS reprogramming, such as small molecule compound, mRNA and Episomal however, the reprogram efficiency is very low [11–13]. Sendai virus (SeV) vector, a cytoplasmic RNA vector, was shown to deliver transgenes efficiently into several cell types and to generate transgene-free iPSCs with high efficiency [14–17]. So SeV vector may be a good choice for the transduction and iPSC reprogramming of old diseased tissue cells.

iPSCs have great potential to differentiate into various cell types [17–19]. Different methods including co-culture systems and growth factors have been used for the differentiation of ESCs or MSCs into NPCs [20–23], and growth factors and hypoxic conditions were used to differentiate iPSCs into NP-like cells [24, 25]. Although different methods for the differentiation of stem cells into NPCs have been reported, the differentiation potential of iPSCs into NP-like cells has not yet been fully studied.

Here, we used the SeV vector encoding OCT3/4, SOX2, KLF4, and c-MYC to reprogram NPCs from degenerated NP tissue collected during surgery, and efficiently generated transgene-free hiPSCs from DDD patients. We were also successful with the induction of iPSCs into NP-like cells. The differentiation potential of NP-like cells is much higher in NP derived iPSCs than that of fibroblast derived iPSCs; NP derived iPSCs have a higher differentiation ratio in hydrogel than in the culture plate. Non-differentiated NP derived iPSCs also showed NPC lineage markers and extra cellular matrix, which indicate their great potential for the study and cellular therapy of DDD.

## RESULTS

### Generation of NPC-derived hiPSCs using Sendai virus vectors

**Figure 1:**
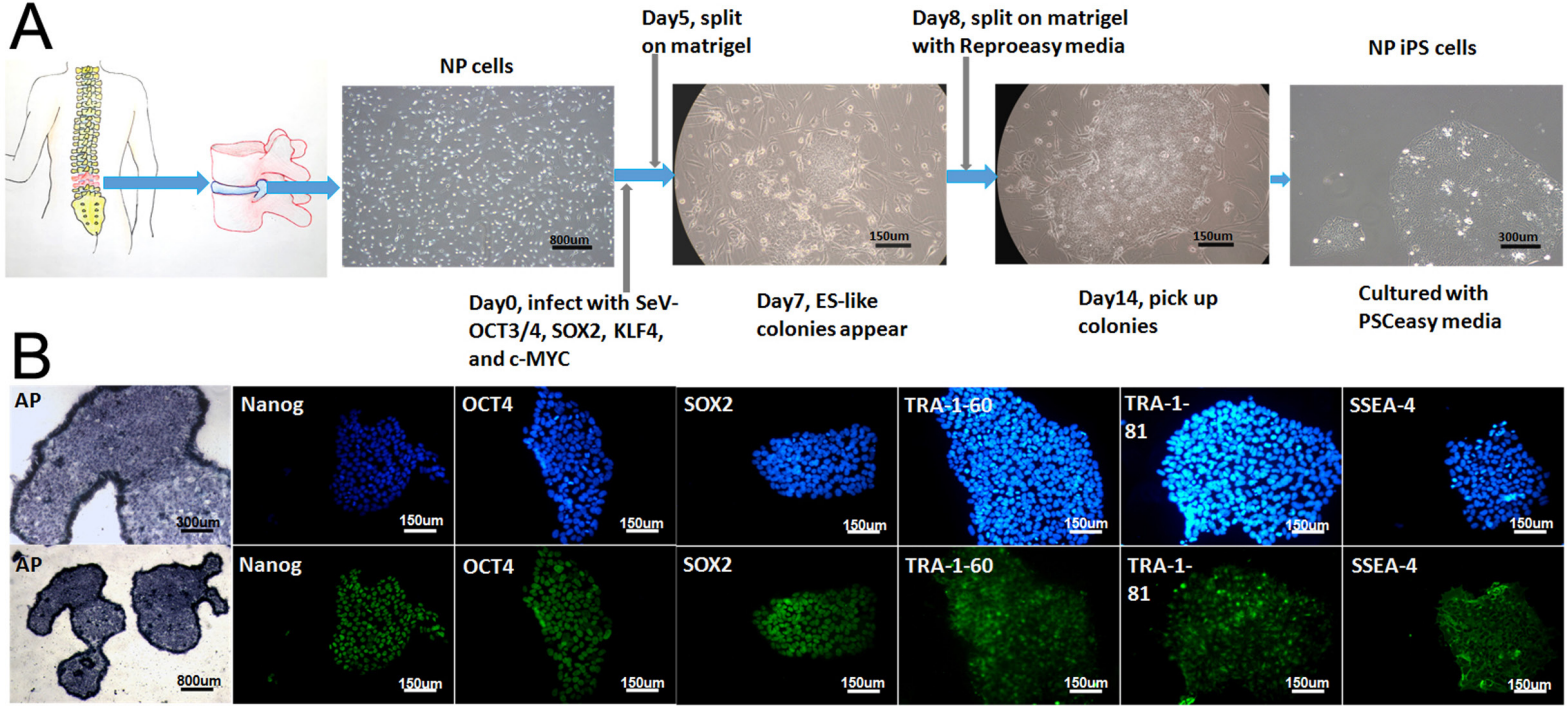
Overview of the NP-iPSC generation protocol and immunostaining of specific markers (**A**) NPCs transduced with SeV expressing human *OCT3/4, SOX2*, *KLF4* and *c-MYC*. NPC derived iPS colonies emerge at 14–28 days after NP cell transduction. (**B**) Staining of Alkaline phosphatase (AP) and pluripotency markers (NANOG, OCT3/4, SOX2, TRA-1–60, TRA-1–81 and SSEA-4) in induced cell colonies

We obtained nucleus pulposus cells from three DDD patients, and then generated NPC derived hiPSC using SeV encoding OCT3/4, SOX2, KLF4, and c-MYC. The schematic of the reprogramming protocol of NPCs by SeV is summarized in Figure 1A. From day 7, some small and loosely packed colonies appeared, that did not show an ESC-like morphology. Between 14 and 28 days, we isolated the packed colonies with similar morphology to ESCs and then these NPC-derived ESC-like colonies were passaged and grown on Matrigel-coated plates.

### Characterization of NP-derived iPSCs

All three NP-derived iPSCs were characterized and has no significant difference, we just randomly chose one of the NP-derived iPSC (female, 44 years old) and showed it's characteristics in the data. To examine the stemness of the NP-derived iPSC, immunostaining was performed to show the positive expression of TRA-1–60, TRA-1–81, SSEA-4, OCT4, SOX2, and NANOG (Figure 1B), and of alkaline phosphatase (Figure 1B).

**Figure 2:**
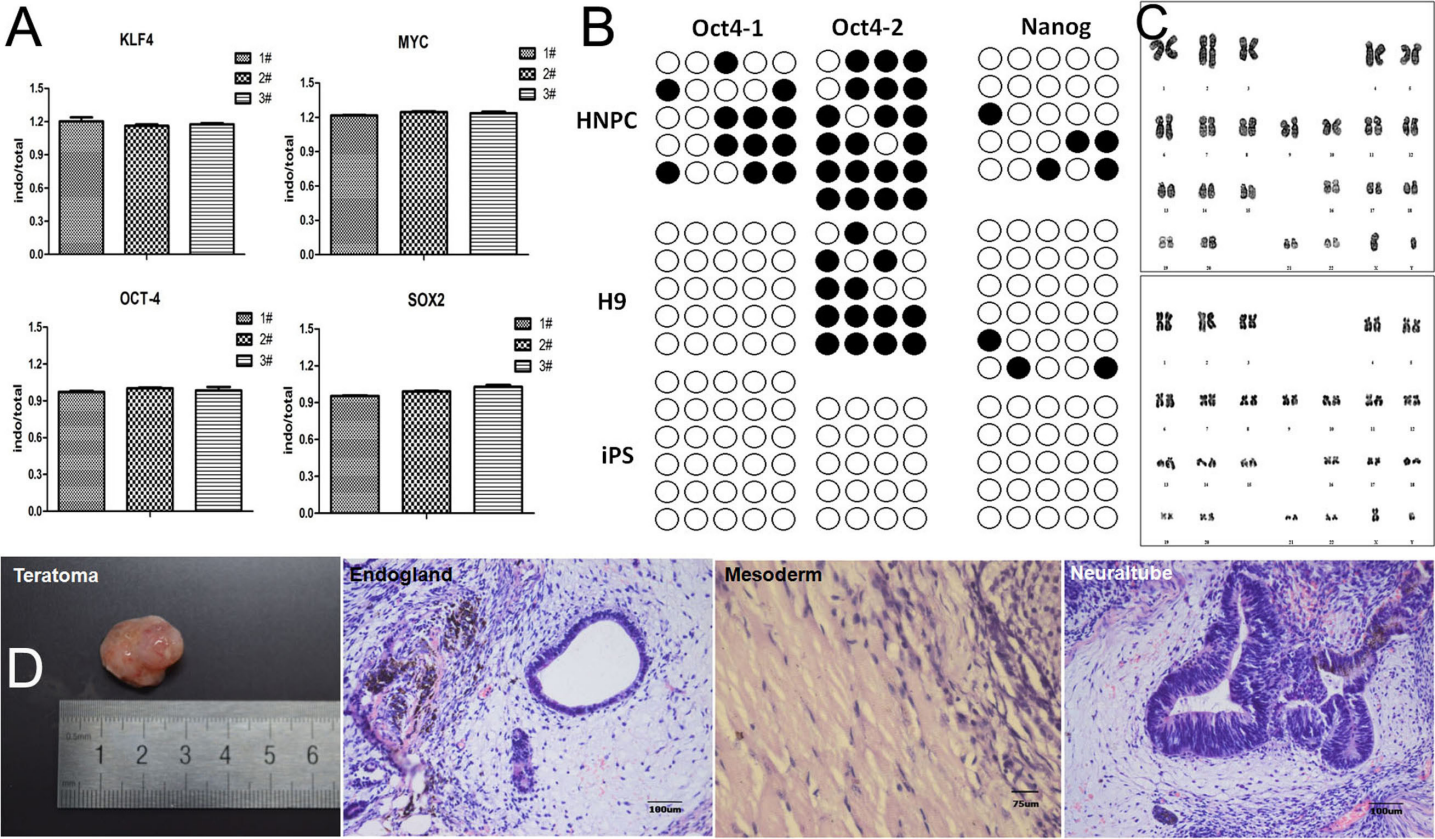
Characterization of iPS colonies generated from NPCs (**A**) Real time polymerase chain reaction (RT-PCR) analysis of endogenous and total genes in induced colonies. 1# and 2# represent NPC-derived iPSCs P4 and P16, 3# represent H9 P38. (**B**) DNA methylation analysis in the promoters of Oct4 and Nanog by bisulfite sequencing. Human NP and H9 cells were used as controls. (**C**) Karyotyping analysis of a representative colony. (**D**) Hematoxylin and eosin (HE) staining of teratomas derived from NP-derived iPSCs.

To monitor the residual amount of SeV in NP-derived iPSCs, we performed real time-PCR analysis for - OCT4, SOX2, c-MYC, and KLF4, global expression analysis of NP-derived iPSC clones (P4 and P16) compared to H9 hESCs showed that NP-derived iPSCs were negative for transduced transgenes after passage 4 (Figure 2A), and NP-derived iPSCs expressed OCT4 and SOX2 at both RNA (Figure 2A) and protein (Figure 1B) levels.

A well known characteristic of ESCs is the low degree of DNA methylation in the promoters of key regulators including Oct4 and Nanog, and many others. DNA methylation of ESC master genes has also been reported to correlate closely with the efficiency of iPS reprogramming. Bisulfite sequencing demonstrated absence of methylation of the endogenous Oct4 and Nanog promoters in our iPSC clones but a high degree of methylation in H9 and NP cells (Figure 2B).

After exceeding 20 passages, NP-derived iPSCs still maintained the normal karyotypes of either 46 XX or 46 XY (Figure 2C).

To study the differentiation potential *in vivo*, NP-derived iPSCs were injected intra-muscularly into NOD-SCID mice, and then the teratomas were histologically analysed (Figure 2D). In the teratomas, we observed the formation of various tissues from the three germ layers; such as neuraltube from ectoderm, endogland from endoderm, and cartilage from mesoderm.

### Differentiation of iPS cells into NP-like cells

**Figure 3:**
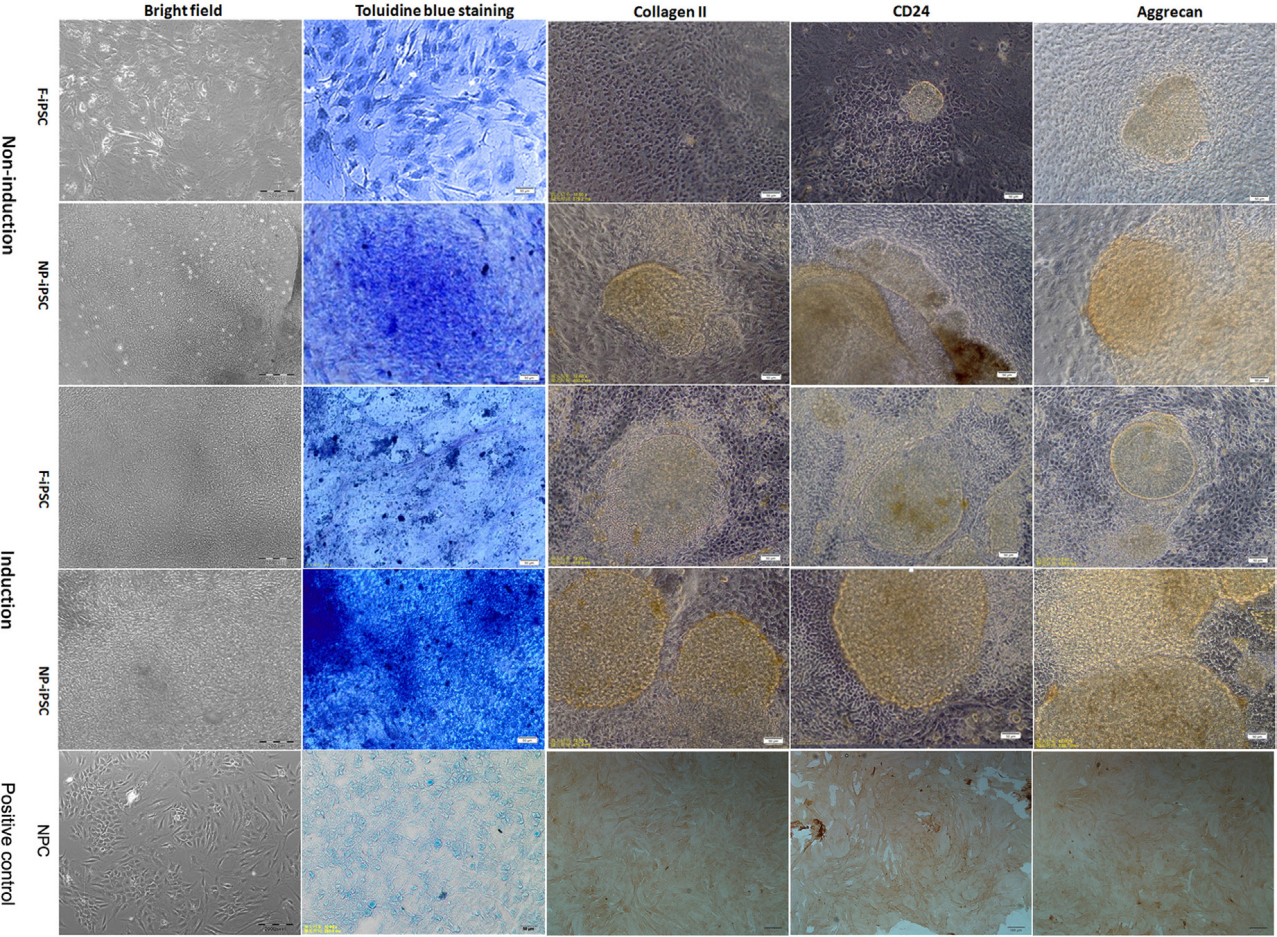
Differentiation analysis of iPSCs Brightfield image showing the morphology of iPSCs 3 weeks post differentiation (magnification 100×, bar = 100 μm). Toluidine blue staining of glycosaminoglycans in representative iPS cell clusters (magnification 200×, bar = 50 μm). Immunostaining of iPS cell cultures for the presence of collagen II, aggrecan, and CD24. NP cells were used as positive control (magnification 100×, bar = 100 μm). Regions of positive staining are shown as brown (magnification 200×, bar = 50 μm). F-iPS: fibroblast derived iPS cells; NP-iPS: NP cells derived iPS cells; NPC: NP cells; Induction: iPSCs induced with NP differentiation media; Non-induction: iPSCs cultured without NP differentiation media.

iPSCs cultured on pre-coated matrigel plates at high density formed large cell clusters which were stable over the duration of differentiation (Figure 3). To verify the production of an NP matrix by iPSCs after differentiation, glycosaminoglycans (GAGs) within the cell clusters were measured by toluidine blue staining (Figure 3). Cells cultured with NP differentiation media synthesize more GAGs than those with nondifferentiation media. NP-derived iPSCs produced significantly higher levels of GAGs than that of fibroblast derived iPSCs, while NP-derived iPSC clusters also showed GAGs expressed when cultured with nondifferentiation media. Cell clusters were immunostained for collagen II, aggrecan, and cluster of differentiation 24 (CD24). Cell clusters cultured in NP differentiation media stained robustly for collagen II, aggrecan, and CD24. However, NP-derived iPSC clusters cultured in basic media also showed weak staining for collagen II, aggrecan, and CD24 (Figure 3). This result, along with Toluidine blue staining, clearly demonstrates that NP-derived iPSCs have higher potential for NP differentiation than fibroblast derived iPSCs.

### Quantitative analysis of NP specific proteins and genes

**Figure 4:**
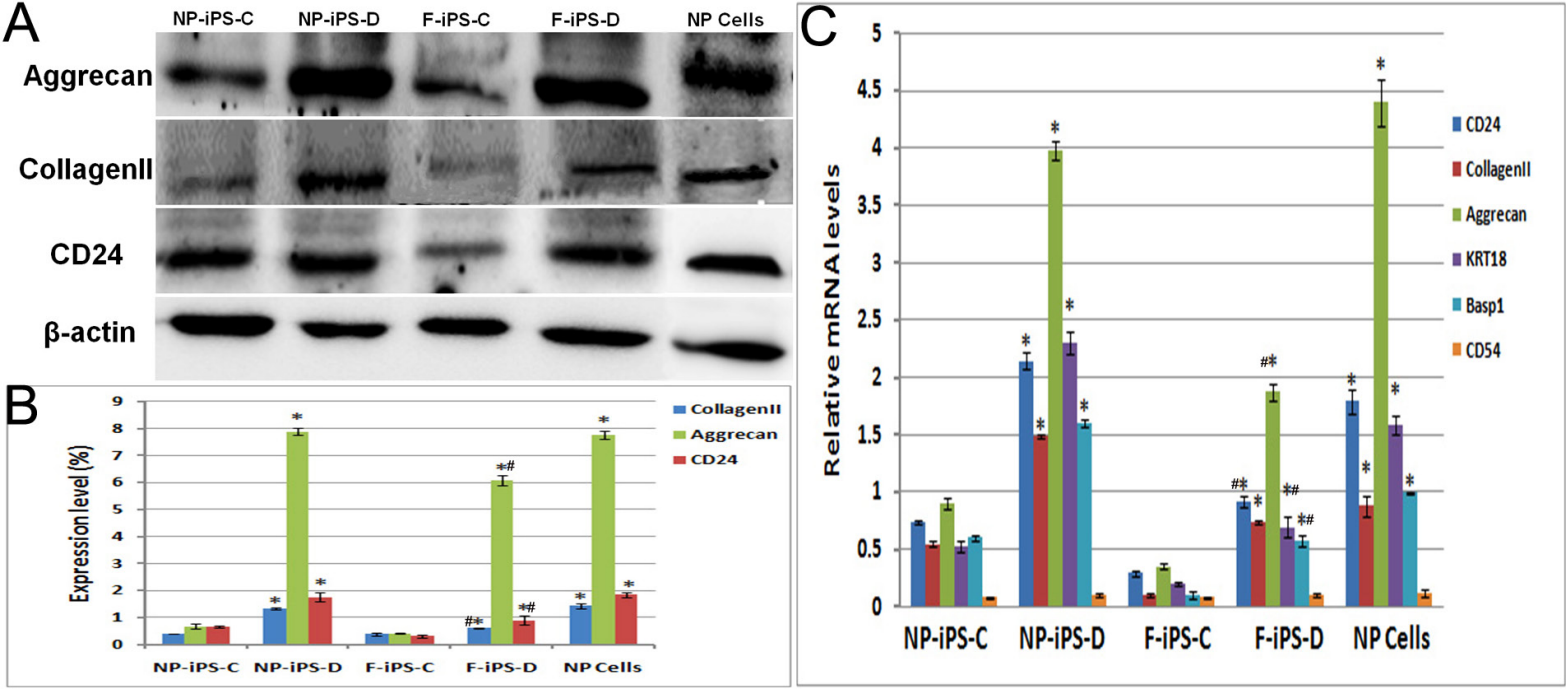
Quantitative analysis of NP specific proteins and genes Protein expressions of collagen II, aggrecan and CD24were analyzed by western blotting. Quantitative analysis was did for collagen II, aggrecan, CD24, KRT18, Basp1 and CD54 gene expression in micromass-cultured iPSCs after 3 weeks differentiation, * compare with undifferentiation group *p < 0.01*, # compare with NP cells *p < 0.05*. F-iPS-C: fibroblast derived iPS cells without differentiation; F-iPS-D: fibroblast derived iPS cells cultured with NP differentiation media; NP-iPS-C: NP cells derived iPS cells without differentiation; NP-iPS-D: NP cells derived iPS cells cultured with NP differentiation media.

Semi-quantitative protein expression by Western blotting showed similar results to immunochemical staining. The expression of collagen II and aggrecan in iPSCs cultured with NP differentiation media were significantly higher post differentiation. Without differentiation, NPC-derived iPSCs had a slight expression of NP ECM, while fibroblast derived iPSCs had almost no NP ECM expression, and without a significant difference between them. After differentiation, the protein expression levels have no significant difference between NPC-derived iPSCs and NP cells, however, the expression levels of fibroblast-derived iPSCs are much lower than NP cells (Figure 4)

Quantitative PCR was used to measure the gene expression profile of CD24, collagen II and aggrecan. Compared to iPSCs cultivated in PSCeasy culture media, iPSCs cultured with NP differentiation media expressed a higher level of collagen II, aggrecan, CD24, KRT18, Basp1 and CD54. NPC-derived iPSCs have similar expression levels with NP cells. In both undifferentiated groups, mRNA expression levels of these specific genes were low and had no significant difference (Figure 4C). This data demonstrates that NPC-derived iPSCs possess greater NP differentiation potential when compared to fibroblast derived iPSCs.

### Differentiation of NP derived iPSCs in hydrogel

**Figure 5:**
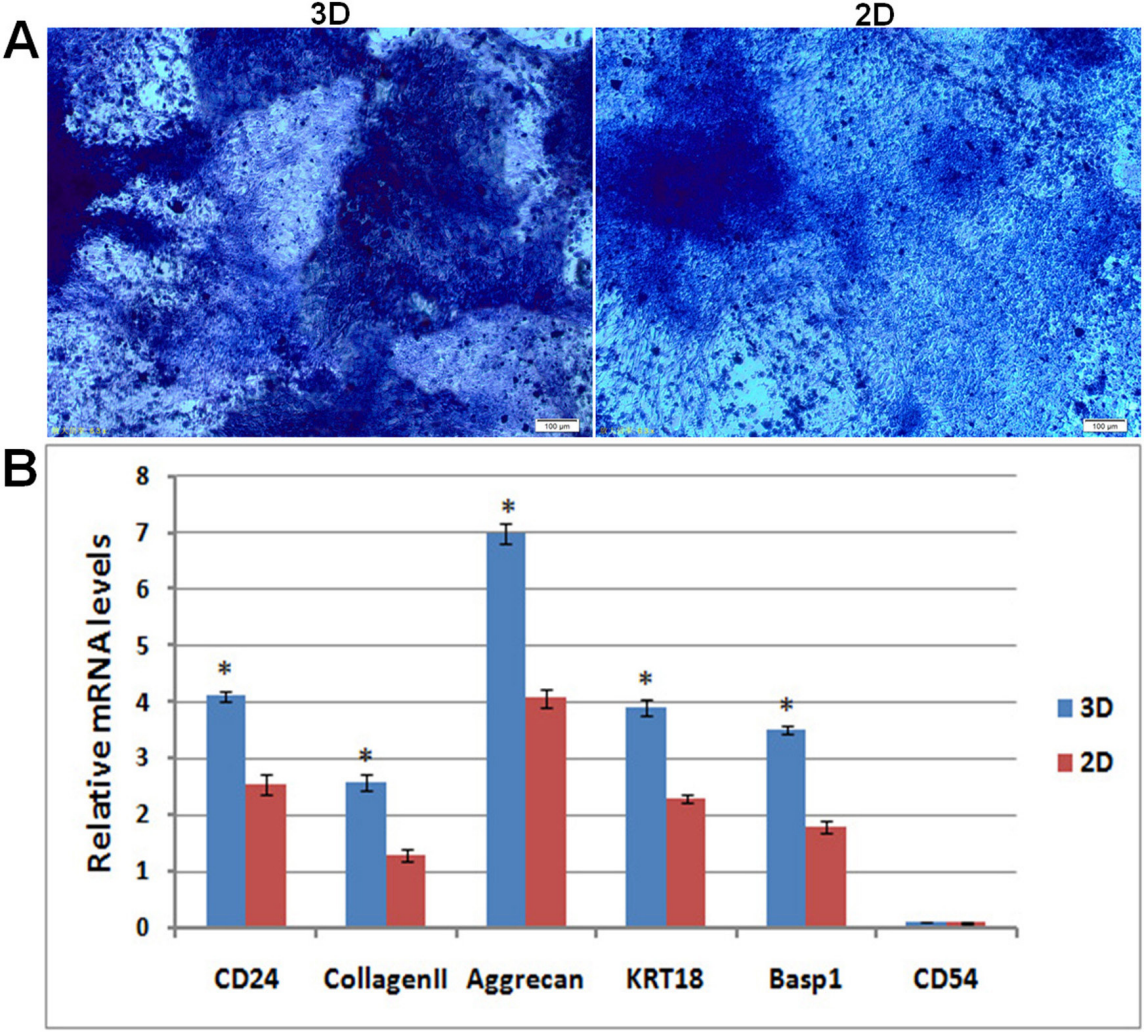
Differentiation analysis of iPSCs in hydrogel Toluidine blue staining of glycosaminoglycans in hydrogel and culture plate (magnification 100×, bar = 100 μm). Quantitative analysis was performed for collagen II, aggrecan, CD24, KRT18, Basp1 and CD54 gene expression in in hydrogel and culture plate, * compared with cells in culture plate. 2D: iPSCs differentiated in culture plate, 3D: iPSCs differentiated in hydrogel.

**Figure 6:**
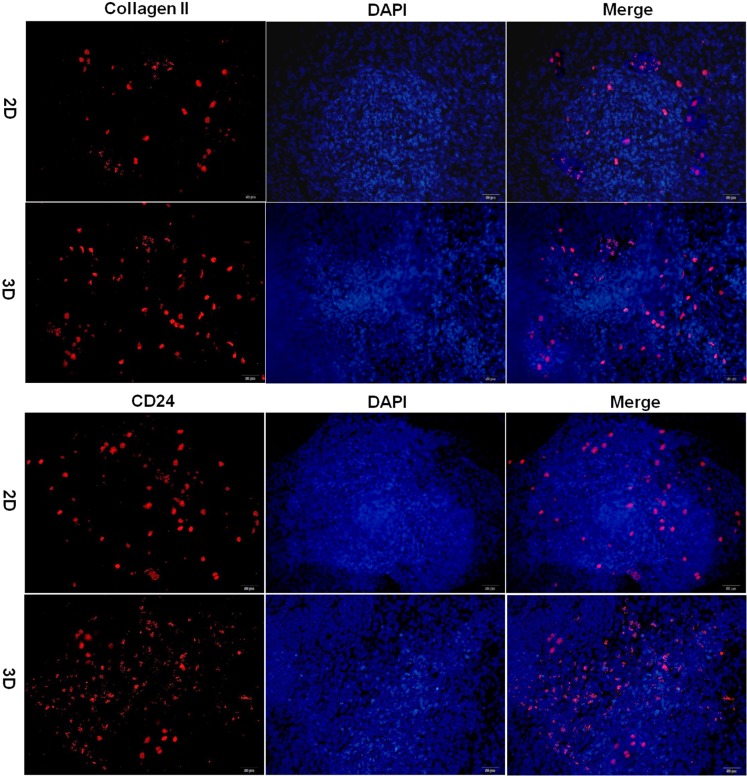
Immunofluorescence analysis of iPSC differentiation in hydrogel Immunofluorescence staining of iPSCs for the presence of collagen II and CD24. Positive staining is shown by red fluorescence, blue fluorescence is for the nucleus (magnification 200×, bar = 50 μm). 2D: iPSCs differentiated in culture plate, 3D: iPSCs differentiated in hydrogel.

After 21 days of differentiation, cell-hydrogel constructs were collected for frozen sections. Sections stained with toluidine blue revealed more extensive accumulation of GAGs with differentiated iPSCs in hydrogel when compared to that in the culture plate (Figure 5A). The real-time PCR results indicated that there are more collagen II, aggrecan, CD24, KRT18, Basp1 and CD54 gene expression in hydrogel than in culture plate (Figure 5B). Immunostaining also demonstrated that there is more collagen II and CD24 expression in hydrogel sections than in culture plates (Figure 6). Positive staining for collagen II and CD24 are shown as red fluorescence, whereas the nucleus is counter stained with DAPI and is shown as blue fluorescence.

## DISCUSSION

The generation of iPSC is a novel method developed in biomedicine. In the past 10 years, original iPSC technology has been modified by improving combinations of reprogramming factors [26, 11], delivery vectors [12, 13, 27], or the cell type to be reprogrammed [5–7]. It is important to find a simple and efficient method to generate patient-specific, viral-free iPSCs without transgene integration from an accessible cell source. Somatic cells can be reprogrammed back iPSCs, which is not only beneficial for understanding the biological mechanisms in chronic and complex diseases (such as DDD), but also serve as a relatively safe cell source for possible therapeutic use. To date, most studies have focused on normal patient tissue, such as skin fibroblast, blood and cord blood [5, 7, 28]. As an easily accessible cell source in DDD patients, nucleus pulposus cell (NPC) from degenerative NP tissue is a better cell source for iPS reprogramming, and subsequent disease study and therapy.

In this study, we have successfully generated transgene-free human iPSCs from NPCs using a simple and efficient method by means of SeV. SeV is an integration free and self-erasable reprogramming system that is not genotoxic, and able to infect a wide range of cell types with high transduction efficiency [10]. NPCs from degenerative NP tissue have a limited ability to proliferate. In our previous studies, we transduced NPCs with constructed retroviruses or lentiviruses, and only a few positive clones were obtained, besides, the incomplete transgene silencing that may have a negative impact on iPSC differentiation [29]. Due to SeV vector's high transduction efficiency without transgene integration, we chose it as the transduction vector for NPC reprogramming. After reprogramming, iPSCs possessed properties of pluripotent stem cells, expressed pluripotency marker genes and proteins, had the ability to form teratomas, had a normal karyotype and completely silenced transgenes after more than 16 passages.

Reprogramming patients’ somatic cells into iPSCs and differentiating them into cells of interest is an excellent tool for studying human diseases. In order to use these patient-specific iPSCs to study DDD, we differentiated these iPSCs into NP-like cells. So far, there are scarcely available protocols for inducing iPSC differentiation into the NP like cell. Chen et al. differentiated iPSCs into the NP-like cell phenotype with native NP tissue culture media under hypoxic conditions, the enriched CD24+ fraction of iPSCs was directed toward an NP-like phenotype without growth factors [21]. Feng et al. differentiated mouse pluripotent stem cells into functional NP cells with transforming growth factor beta 1 (TGF-β1) [22]. Both protocols resulted in the expression of NP cell lineage markers and NP functional characteristics. In our previous study, we differentiated firoblast-derived iPSC into the chondrocyte-like cell with TGF-β1 to treat osteoarthritic rats (data to be published soon). In this study, we differentiated NP-derived iPSCs into NP-like cells with modified differentiation media, and compared its differentiation ability with fibroblast derived iPSCs. Both differentiated iPSCs maintained a clustered morphology when cultured with theMatrigel substrate, whereby the soft stiffness of Matrigel may simulate that of the native NP [30], which contributed to the directed differentiation of iPSCs into NPCs.

iPSCs were induced to differentiate into NP-like cells, and this was confirmed by the expression of NP cell markers and the ability of the cells to synthesise glycosaminoglycans, type II collagen and aggrecan. Collagen II and aggrecan are key molecules produced by NP cells [31]. Group of Orthopedic Research Society recommended the stabilized expression of HIF-1, Glut-1, aggrecan/collagen, Shh, KRT18, CA12, Basp1 and CD24 as the healthy NP phenotypic markers [32], CD24 as a marker of the NP precursor cell, closely identified within notochord [33, 34], which may be served as a human NP phenotypic marker in the early stage of disc development. CD54 expression were very faint in younger NP tissue, they showed stronger expression in aged NP tissue, CD54 may be used as a biomarker to evaluate the inflammation-associated disc degeneration [35], and also can be used as a marker for inflammation microenvironment during disc development. All these marker genes increased significantly after differentiation except CD54, which indicated that these are no inflammation reaction in the differentiated NP-like cells. The expression patterns of NP-like markers and the clustering morphology in our differentiated iPSCs were similar to that observed in native human NP tissue. With our differentiation condition, both NP derived iPSCs and fibroblast derived iPSCs showed an NP like characteristic. NP derived iPSCs without differentiation also showed the clustering morphology, glycosaminoglycan positive staining and NP-like markers, to a greater degree compared to fibroblast derived iPSCs. This data demonstrates the high NP differentiation potential of NP derived iPSCs, which may be in part be due to the memory of the original NP tissue. In our differentiation system, the interaction of matrigel substrate stiffness and growth factor supplementation seems to be important for promoting iPSC differentiation into NP-like cells *in vitro*.

In summary, we have successfully generated iPSCs from the NP tissue of DDD patients with SeV. iPSC reprogramming from DDD patients’ NP tissue may retain the memory of NP specific characteristics, which may promote the directed differentiation of iPSC into NP cell, and the 3D differentiation system can promote the differentiation of iPSC into NP-like cell. iPSC generated from DDD patients can therefore be a disease model for the study of its biological mechanisms, or can serve as a relatively safe cell source for possible therapeutic use in DDD treatment in the future.

## MATERIALS AND METHODS

### Nucleus pulposus cell isolation and culture

The study was approved by the Medical Ethical Committee and the Animal Ethical and Welfare Committee of Shenzhen University. All patients provided written informed consent and the degenerated nucleus pulposus from 3 DDD patients (38–50 years old, Pfirrman classification grade 6) was obtained during surgery. The nucleus pulposus cells were isolated by digestion with 0.1% collagenase (Invitrogen) and 0.25% trypsin (Invitrogen) for about 20 min at 37°C; the digestion buffer was carefully collected and transferred to centrifuge tubes with culture medium (Dulbecco's Modified Eagle Medium-F12 (DMEM-F12, Gibco) containing 10% fetal bovine serum (FBS, Gibco)). The digested mixture was centrifuged at 1500 rpm for 10 min, the supernatant was carefully removed and then the trypsin-collagenase was re-added to the remnant tissue. The above steps were repeated two to three times, until the nucleus pulposus tissue was digested completely. Cell pellets were then resuspended in culture medium and plated in T25 flasks and changed media every 3 days..

### Generation of iPSCs from human nucleus pulposus cell

Reprogramming of nucleus pulposus cell (NPC, passage 1) into iPSC was performed 5 days after initial culture using SeV encoding OCT3/4, SOX2, KLF4, and c-MYC (CytoTune 2.0, Invitrogen). The transduced cells were trypsinized with 0.25% trypsin, resuspended in the same medium, and plated onto a 6-well plate that was pre-coated with matrigel (BD Biosciences). On the next day, medium was refreshed and then. two days later, cells were trypsinized and passaged onto two 10cm matrigel-coated culture dishes. The cultures were maintained in Reproeasy culture medium with factors (Cellapy, China). Finally, iPSC colonies were manually isolated based on morphology between day 14 to day 28 post-infection and were maintained on plates coated with Matrigel in PSCeasy culture medium with factors. All three NPCs were reprogrammed and characteriazed.

### Gene analysis and protein expression

Total RNA from NP-derived iPSCs (passage 4 and 16) was isolated with TRIzol (Invitrogen), and RNA was treated with DNase I (Invitrogen). cDNA was synthesized using Superscript II reverse transcriptase (Invitrogen). Realtime-PCR analysis for hESC marker genes OCT4, SOX2, NANOG and KLF4 was performed. hESC H9 were used as control. Endogenous and total primers of OCT4, SOX2, NANOG and KLF4 were showed in Table 1. cDNA samples were also subjected to TaqMan Human Stem Cell Pluripotency Array (Life Technologies) to compare with the endogenous and total gene expression.

**Table 1:** Endogenous and total primers of OCT4, SOX2, NANOG and KLF4

Target	Sequence (5′---3′)	Product sizes(bp)
KLF4 Endo	cggacctacttactcgcctt	192
	gcaactgaaaccccaagtcc	
KLF4 Total	tctccaattcgctgacccat	139
	tcgaagtggataggctaggc	
MYC Endo	catccacgaaactttgccca	152
	tcctgggcgaagagactttc	
MYC Total	aacacacaacgtcttggagc	193
	gacttctcctgaacaacgcc	
OCT-4 Endo	ctgctgggtctcctttctca	120
	catgaggagccagggaaagg	
OCT-4 Total	cgagaggattttgaggctgc	126
	agtgacgtgacatgaggagc	
SOX2 Endo	tcaggagttgtcaaggcaga	124
	tccctctcttcaaactcggg	
SOX2 Total	agctcgcagacctacatgaa	151
	atggagaaggagggtgaggt	

Immunocytochemistry of NP-derived hiPSCs (passage 13) was performed using the following antibodies based on the manufacturers’ protocols: Anti-human TRA-1-60 (StainAlive TRA-1-60, Stemgent), Anti-human TRA-1-81 (StainAlive TRA-1-60, Stemgent), anti-human SSEA-4 (Santa Cruz Biotechnology), anti-human NANOG (Abcam), anti-human OCT3/4 (Santa Cruz Biotechnology) and anti-human SOX2 (Santa Cruz Biotechnology). Cells were examined under a Zeiss Imager Z1 fluorescence microscope and photographed with the AxioVision software.

### Teratoma formation assay for pluripotency

To test the pluripotency of NP-derived iPSC *in vivo*, NP-derived iPSCs (passage 16) were harvested from four wells of the six-well plates at 80% confluence using 0.5mM EDTA and resuspended in 100 uL of hiPSC medium. Cells were injected intramuscularly into nonobese diabetic combined severe immunodeficient (NOD-SCID) mice. Tumors were excised 15 weeks post injection, fixed in 10% formalin, and embedded in frozen embedding medium, sectioned and then stained with Hematoxylin & Eosin (H&E).

### Bisulfite genomic sequencing

Bisulfite treatment was performed using the MethylCode Bisulfite Conversion Kit (Invitrogen) according to the manufacturer's recommendations. PCR primers for Oct4 and Nanog promoters were included as follows:

(OCT4-1-F (bp1): 5′-GGATGTTATTAAGATGAAG ATAGTTGG-3′;

OCT4-1-R (bp406): 5′-CCTAAACTCCCCTTCAA AATCTATT-3′;

OCT4-2-F (bp1): 5′-TAGTTGGGATGTGTAGAGT TTGAGA-3′;

OCT4-2-R (bp260): 5′-TAAACCAAAACAATCC TTCTACTCC-3′;

NANOG-F (bp3776): 5′-GGAGTAGAGTGTAGA GGAGAATGAGTTA-3′;

NANOG-R (bp4107C): 5′-CTAACTCTTTAACTTC TTCCCAAATC-3′.

PCR products were cloned into pMD18-T(Takara), and 5-6 randomly selected clones for each gene were sequenced with M13 forward and M13 reverse primers.

### Determination of karyotypes

For karyotype analysis, NP-derived iPSCs (passage 16) were treated with Demecolcine (0.07 mg/mL final concentration, Sigma-Aldrich) for 4 h. The cells were subsequently centrifuged and resuspended in 10 mL KCl solution (75 mM), followed by three rounds of fixation using an ice-cold methanol: glacial acetic acid (3:1) solution. The fixed cells were washed at least twice with 10 mL fixative solution before applying to chilled slides. Slides prepared for chromosome analysis were dried and treated with 0.0025% trypsin for 5 min and stained with Giemsa (1:10, Sigma-Aldrich,) for 5–10 min and finally, a karyogram was produced for two cells per clonal line.

### Differentiation of iPSC into NP-like cell

Fibroblast derived iPSCs were generated and identified by the same method. fibroblasts were obtained from osteoarthritis patient, and fibroblast derived iPSCs were characterized [36]. When we differentiate NPC derived iPSCs into NP-like cells, human fibroblast derived iPSCs were used as a control. To differentiate NPC derived iPSC into NP-like cells, iPSCs at a high density (1 × 10^7^ cells/ml) were dropped into the centre of a culture plate pre-coated with Matrigel, allowed to adhere for 1h, and then differentiation was induced with an NP differentiation medium (high glucose DMEM supplemented with 10ng/mL TGF-β1, 100 nM dexamethasone, 50 μg/mL ascorbic acid 2-phosphate, 1% ITS, 100U/mL penicillin, and 100 mg/mL streptomycin, all from Life Technologies) at 37°C in a 5% CO2 atmosphere. The differentiation media was changed twice per week and cells cultured in high glucose DMEM with 10% FBS served as a negative control (undifferentiatied cells). At Day 21, the cell pellets were then evaluated as described below.

### Immunohistochemical staining

Prior to immunohistochemical staining, undifferentiated iPSCs and their differentiated derivatives, as well as NP positive control cells, were firstly fixed in cold acetone for 30 min at 4°C. Cells were then rinsed with PBS three times and blocked in 10% normal goat serum for 20 min. Samples were incubated with primary antibodies (Anti-Collagen II, Anti-Aggrecan, Anti-CD24, Abcam) overnight at 4°C, then rinsed with PBS three times, incubated with horseradish peroxidase (HRP)-conjugated secondary antibodies (Invitrogen) for 2h at room temperature, rinsed again with PBS three times and then developed by use of the DAB kit. For the analysis of glycosaminoglycan, cells were fixed with formaldehyde, and stained with Toluidine blue. All immages were captured using an IX-71 fluorescence microscope (Olympus,Japan).

### Specific protein expression by Western blotting

After differentiation, the cells were collected and lysed in Tris–HCl buffer 0.05 mol/L (pH 8.0) containing 0.15 mol/L sodium chloride, 0.02% sodium azide, 0.1% SDS, 1% nonidet P (NP-40), 11 μg/mL aprotinin, and 0.1 mol/L phenylmethyl sulfonylfluoride (PMSF) (all reagents from Sigma Aldrich, USA). NP cells were used as positive control. The cell lysates were centrifuged at 12,000×g for 5 min at 4°C. SDS-PAGE was performed using 100μg total protein aliquots together with prestained molecular weight standards. Proteins were transferred onto nitrocellulose membranes, and then blocked with dried skimmed milk powder in Tween PBS-buffered saline (PBS-T) for 1 h at room temperature. Membranes were probed with primaryantibodies (Anti-CollagenI I, Anti-Aggrecan, Anti-CD24, 1:1000, Abcam) in PBS-T overnight at 4°Cand then horseradish peroxidase-conjugated secondary antibodies were incubated with the protein blots for 1 h at room temperature. Immunoreactive protein was detected using ECL chemiluminescence and images captured by the LAS-3000 imaging system (Fujifilm).

### Analysis of mRNA expression levels

Total RNA from the differentiated cells and NP positive control cells were obtained using Trizol (Invitrogen). The RNA was reverse transcribed to complementary DNA (cDNA) using the First Strand cDNA kit (Takara) following the manufacturer's protocol. Quantitative polymerase chain reaction (qPCR) analysis was then performed using the Quantitect SYBR Green PCR master mix (Takara). Standard curves were generated, and the quantities of each transcript were normalized to β-actin that was used as an internal control.

### Differentiation of NP derived iPSCs in hydrogel

Because NP cells live in a three dimensional (3D) environment, NP derived iPSCs were differentiated in chitosan-hyaluronic acid hydrogel. Briefly, 1ml 2% chitosan and 2% hyaluronic acid were mixed with 2×106 cells, and seeded into a 24 well plate at 37°C for the development of the cell-hydrogel complex. After one week of cultivation with PSCeasy culture medium, differentiation media was then used for 21 days. The same number of iPSCs cultured at the same conditions in a 24 well plate was used as control. Toluidine blue staining, immunostaining for CD24 and collagen II, and real-time PCR were performed 21 days post-differentiation.

### Statistical analysis

Statistical evaluation was performed using analysis of variance followed by a post hoc Student's *t-test*. A *p value* of less than 0.05 was considered significant andthe data is expressed as mean ± SD.

H&E, Hematoxylin & Eosin; PMSF, phenylmethyl sulfonylfluoride; PBS-T, PBS-buffered saline; qPCR, quantitative polymerase chain reaction; GAGs, glycosaminoglycans; CD24, cluster of differentiation 24.

## Abbreviations

NP: nucleus pulposus
NPC: nucleus pulposus cell
DDD: disc degenerative disease
iPSCs: induced pluripotent stem cells
IVD: intervertebral disc
MSCs: mesenchamal stem cells
ESCs: embryonic stem cells
SeV: Sendai virus
DMEM-F12: Dulbecco's Modified Eagle Medium-F12
HPRT: Hypoxanthine guanine phosphoribosyl transferase
NOD-SCID: nonobese diabetic severe combined immunodeficient;

## References

[1] Zhang Y, Xiong C, Chan C, Sakai D, Chan D (2015). Changes in Nucleus Pulposus Cell Pools in “Healer” Mice for the Repair of Intervertebral Disc Degeneration. Global Spine J.

[2] Kim DH, Martin JT, Elliott DM, Smith LJ, Mauck RL (2015). Phenotypic stability, matrix elaboration and functional maturation of nucleus pulposus cells encapsulated in photocrosslinkable hyaluronic acid hydrogels. Acta Biomaterialia.

[3] Oehme D, Goldschlager T, Ghosh P, Rosenfeld JV, Jenkin G (2015). Cell-Based Therapies Used to Treat Lumbar Degenerative Disc Disease: A Systematic Review of Animal Studies and Human Clinical Trials. Stem Cell International.

[4] Lv F, Leung V, Cheung K (2016). Cell-based Therapies for Degenerative Disc Diseases. Operative Techniques in Orthopaedics.

[5] Takahashi K, Tanabe K, Ohnuki M, Narita M, Ichisaka T, Tomoda K, Yamanaka S (2007). Induction of pluripotent stem cells from adult human fibroblasts by defined factors. Cell.

[6] Aoi T, Yae K, Nakagawa M, Ichisaka T, Okita K, Takahashi K, Chiba T, Yamanaka S (2008). Generation of pluripotent stem cells from adult mouse liver and stomach cells. Science.

[7] Nishishita N, Takenaka C, Fusaki N, Kawamata S (2011). Generation of induced pluripotent stem cells from human cord blood. J Stem Cells.

[8] Seki T, Yuasa S, Fukuda K (2012). Generation of induced pluripotent stem cells from a small amount of human peripheral blood using a combination of activated T cells and Sendai virus. Nature protocols.

[9] Gonzalez F, Boue S, Izpisua Belmonte JC (2011). Methods for making induced pluripotent stem cells: Reprogramming a la carte. Nat Rev Genet.

[10] Chen IP, Fukuda K, Fusaki N, Iida A, Hasegawa M, Lichtler A, Reichenberger EJ (2013). Induced Pluripotent Stem Cell Reprogramming by Integration-Free Sendai Virus Vectors from Peripheral Blood of Patients with Craniometaphyseal Dysplasia. Cellular Reprogramming.

[11] Fujie Y, Fusaki N, Katayama T, Hamasaki M, Soejima Y, Soga M, Ban H, Hasegawa M, Yamashita S, Kimura S, Suzuki S, Matsuzawa T, Akari H (2014). New type of Sendai virus vector provides transgene-free iPS cells derived from chimpanzee blood. Plos One.

[12] Requena J, Palomo AB, Sal MF, Christodoulou J, Canals JM, Edel MJ (2014). The Future Challenges for the Clinical Application of Reprogrammed Cells. Human Genet Embryol.

[13] Itoh M, Kawagoe S, Okano HJ, Nakagawa H (2016). Integration-free T cell-derived human induced pluripotent stem cells (iPSCs) from a healthy individual: WT-iPSC2. Stem Cell Research.

[14] Morita A, Soga K, Nakayama H, Ishida T, Kawanishi S, Sato EF (2015). Neuronal differentiation of human iPS cells induced by baicalin via regulation of bHLH gene expression. Biochemical and Biophysical Research Communications.

[15] Aikawa N, Suzuki Y, Takaba K (2015). A Simple Protocol for the Myocardial Differentiation of Human iPS Cells. Biological & Pharmaceutical Bulletin.

[16] Tasnim F, Phan D, Toh YC, Yu H (2015). Cost-effective differentiation of hepatocyte-like cells from human pluripotent stem cells using small molecules. Biomaterials.

[17] Stoyanov JV, Gantenbein-Ritter B, Bertolo A, Aebli N, Baur M, Alini M, Grad S (2010). Role of hypoxia and growth and differentiation factor-5 on differentiation of human mesenchymal stem cells towards intervertebral nucleus pulposus-like cells. European Cells & Materials.

[18] Hu X, Zhou Y, Zheng X, Tian N, Xu C, Wu W, Li F, Zhu S, Zheng Y, Xue E, Yu Y, Zhang X, Xu H (2014). Differentiation of menstrual blood-derived stem cells toward nucleus pulposus-like cells in a coculture system with nucleus pulposus cells. Spine.

[19] Zhou X, Tao Y, Wang J, Liang C, Wang J, Li H, Chen Q (2014). Roles of FGF-2 and TGF-beta/FGF-2 on differentiation of human mesenchymal stem cells towards nucleus pulposus-like phenotype. Growth Factors.

[20] Zhou X, Tao Y, Liang C, Zhang Y, Li H, Chen Q (2015). BMP3 Alone and Together with TGF-β Promote the Differentiation of Human Mesenchymal Stem Cells into a Nucleus Pulposus-Like Phenotype. Int J Mol Sci.

[21] Chen J, Lee EJ, Jing L, Christoforou N, Leong KW, Setton LA (2013). Differentiation of Mouse Induced Pluripotent Stem Cells (iPSCs) into Nucleus Pulposus-Like Cells In Vitro. PLoS One.

[22] Liu K, Chen Z, Luo XW, Song GQ, Wang P, Li XD, Zhao M, Han XW, Bai YG, Yang ZL, Feng G (2015). Determination of the potential of induced pluripotent stem cells to differentiate into mouse nucleus pulposus cells in vitro. Genetics and Molecular Research.

[23] Heng JC, Feng B, Han J, Jiang J, Kraus P, Ng JH, Orlov YL, Huss M, Yang L, Lufkin T (2010). The nuclear receptor Nr5a2 can replace Oct4 in the reprogramming of murine somatic cells to pluripotent cells. Cell Stem Cell.

[24] Hou P, Li Y, Zhang X, Liu C, Guan J, Li H, Zhao T, Ye J, Yang W, Liu K (2013). Pluripotent stem cells induced from mouse somatic cells by small-molecule compounds. Science.

[25] Warren L, Manos PD, Ahfeldt T, Loh YH, Li H, Lau F, Ebina W, Mandal PK, Smith ZD, Meissner A (2010). Highly efficient reprogramming to pluripotency and directed differentiation of human cells with synthetic modified mRNA. Cell Stem Cell.

[26] Park HY, Noh EH, Chung HM, Kang MJ, Kim EY, Park SP (2012). Efficient generation of virus-free iPS cells using liposomal magnetofection. PLoS One.

[27] Lieu PT (2015). Reprogramming of Human Fibroblasts with Non-integrating RNA Virus on Feeder-Free or Xeno-Free Conditions. Methods in Molecular Biology.

[28] Quintana-Bustamante O, Segovia JC (2016). Generation of Patient-Specific induced Pluripotent Stem Cell from Peripheral Blood Mononuclear Cells by Sendai Reprogramming Vectors. Methods in Molecular Biology.

[29] Maherali N, Hochedlinger K (2008). Guidelines and techniques for the generation of induced pluripotent stem cells. Cell Stem Cell.

[30] Gilchrist CL, Darling EM, Chen J, Setton LA (2011). Extracellular Matrix Ligand Stiffness Modulate Immature Nucleus Pulposus Cell-Cell Interactions.

[31] Lin Y, Yue B, Xiang H, Liu Y, Ma X, Chen B (2016). Survivin is expressed in degenerated nucleus pulposus cells and is involved in proliferation and the prevention of apoptosis in vitro. Molecular Medicine Reports.

[32] Risbud MV, Schoepflin ZR, Mwale F, Kandel RA, Grad S, Iatridis JC, Sakai D, Hoyland JA (2015). Defining the phenotype of young healthy nucleus pulposus cells: recommendations of the Spine Research Interest Group at the 2014 annual ORS meeting. J Orthop Res.

[33] McCann MR, Tamplin OJ, Rossant J, Seguin CA (2012). Tracing notochord-derived cells using a Noto-cre mouse: implications for intervertebral disc development. Dis Model Mech.

[34] Maier JA, Lo Y, Harfe BD (2013). Foxa1 and Foxa2 are required for formation of the intervertebral discs. PLoS One.

[35] Tang X, Jing L, Richardson WJ, Isaacs RE, Fitch RD, Brown CR, Erickson MM, Setton LA, Chen J (2016). Identifying molecular phenotype of nucleus pulposus cells in human intervertebral disc with aging and degeneration. J Orthop Res.

[36] Zhu Y, Wu X, Liang Y, Gu H, Song K, Zou X, Zhou G (2016). Repair of cartilage defects in osteoarthritis rats with induced pluripotent stem cell derived chondrocytes. BMC Biotechnol.

